# A genome-wide association study of anorexia nervosa suggests a risk locus implicated in dysregulated leptin signaling

**DOI:** 10.1038/s41598-017-01674-8

**Published:** 2017-06-19

**Authors:** Dong Li, Xiao Chang, John J. Connolly, Lifeng Tian, Yichuan Liu, Elizabeth J. Bhoj, Nora Robinson, Debra Abrams, Yun R. Li, Jonathan P. Bradfield, Cecilia E. Kim, Jin Li, Fengxiang Wang, James Snyder, Maria Lemma, Cuiping Hou, Zhi Wei, Yiran Guo, Haijun Qiu, Frank D. Mentch, Kelly A. Thomas, Rosetta M. Chiavacci, Roger Cone, Bingshan Li, Patrick A. Sleiman, Eating Disorders Working Group of the Psychiatric Genomics Consortium, Vesna Boraska Perica, Christopher S. Franklin, James A. B. Floyd, Laura M. Thornton, Laura M. Huckins, Lorraine Southam, N. William Rayner, Ioanna Tachmazidou, Kelly L. Klump, Janet Treasure, Ulrike Schmidt, Federica Tozzi, Kirsty Kiezebrink, Johannes Hebebrand, Philip Gorwood, Roger A. H. Adan, Martien J. H. Kas, Angela Favaro, Paolo Santonastaso, Fernando Fernánde-Aranda, Monica Gratacos, Filip Rybakowski, Monika Dmitrzak-Weglarz, Jaakko Kaprio, Anna Keski-Rahkonen, Anu Raevuori-Helkamaa, Eric F. Van Furth, Margarita C. T. Slof-Op’t Landt, James I. Hudson, Ted Reichborn-Kjennerud, Gun Peggy S. Knudsen, Palmiero Monteleone, Allan S. Kaplan, Andreas Karwautz, Wade H. Berrettini, Nicholas J. Schork, Tetsuya Ando, Hidetoshi Inoko, Tõnu Esko, Krista Fischer, Katrin Männik, Andres Metspalu, Jessica H. Baker, Janiece E. DeSocio, Christopher E. Hilliard, Julie K. O’Toole, Jacques Pantel, Jin P. Szatkiewicz, Stephanie Zerwas, Oliver S. P. Davis, Sietske Helder, Katharina Bühren, Roland Burghardt, Martina de Zwaan, Karin Egberts, Stefan Ehrlich, Beate Herpertz-Dahlmann, Wolfgang Herzog, Hartmut Imgart, André Scherag, Stephan Zipfel, Claudette Boni, Nicolas Ramoz, Audrey Versini, Unna N. Danner, Judith Hendriks, Bobby P. C. Koeleman, Roel A. Ophoff, Eric Strengman, Annemarie A. van Elburg, Alice Bruson, Maurizio Clementi, Daniela Degortes, Monica Forzan, Elena Tenconi, Elisa Docampo, Geòrgia Escaramís, Susana Jiménez-Murcia, Jolanta Lissowska, Andrzej Rajewski, Neonila Szeszenia-Dabrowska, Agnieszka Slopien, Joanna Hauser, Leila Karhunen, Ingrid Meulenbelt, P. Eline Slagboom, Alfonso Tortorella, Mario Maj, George Dedoussis, Dimitris Dikeos, Fragiskos Gonidakis, Konstantinos Tziouvas, Artemis Tsitsika, Hana Papezova, Lenka Slachtova, Debora Martaskova, James L. Kennedy, Robert D. Levitan, Zeynep Yilmaz, Julia Huemer, Doris Koubek, Elisabeth Merl, Gudrun Wagner, Paul Lichtenstein, Gerome Breen, Sarah Cohen-Woods, Anne Farmer, Peter McGuffin, Sven Cichon, Ina Giegling, Stefan Herms, Dan Rujescu, Stefan Schreiber, H-Erich Wichmann, Christian Dina, Rob Sladek, Giovanni Gambaro, Nicole Soranzo, Antonio Julia, Sara Marsal, Raquel Rabionet, Valerie Gaborieau, Danielle M. Dick, Aarno Palotie, Samuli Ripatti, Elisabeth Widén, Ole A. Andreassen, Thomas Espeseth, Astri Lundervold, Ivar Reinvang, Vidar M. Steen, Stephanie Le Hellard, Morten Mattingsdal, Ioanna Ntalla, Vladimir Bencko, Lenka Foretova, Vladimir Janout, Marie Navratilova, Steven Gallinger, Dalila Pinto, Stephen W. Scherer, Harald Aschauer, Laura Carlberg, Alexandra Schosser, Lars Alfredsson, Bo Ding, Lars Klareskog, Leonid Padyukov, Chris Finan, Gursharan Kalsi, Marion Roberts, Jeff C. Barrett, Xavier Estivill, Anke Hinney, Patrick F. Sullivan, Eleftheria Zeggini, Cynthia M. Bulik, Price Foundation Collaborative Group, Harry Brandt, Steve Crawford, Scott Crow, Manfred M. Fichter, Katherine A. Halmi, Craig Johnson, Allan S. Kaplan, Maria C. La Via, James Mitchell, Michael Strober, Alessandro Rotondo, Janet Treasure, D. Blake Woodside, Cynthia M. Bulik, Pamela K. Keel, Kelly L. Klump, Lisa Lilenfeld, Laura M. Thornton, Andrew W. Bergen, Wade Berrettini, Walter Kaye, Pierre Magistretti, Hakon Hakonarson

**Affiliations:** 1 Center for Applied Genomics, Children’s Hospital of Philadelphia, Philadelphia, PA USA; 2 Department of Molecular Physiology and Biophysics, Vanderbilt University, Nashville, TN USA; 3 Department of Human Genetics, Children’s Hospital of Philadelphia, Philadelphia, PA USA; 4 Department of Pediatrics, The Perelman School of Medicine, University of Pennsylvania, Philadelphia, PA USA; 5 Present Address: Department of Molecular and Integrative Physiology, University of Michigan, Ann Arbor, MI USA; 14 Health Services Research Unit, University of Aberdeen, Aberdeen, UK; 6 Wellcome Trust Sanger Institute, Wellcome Trust Genome Campus, Hinxton, Cambridge UK; 7 University of Split School of Medicine, Split, Croatia; 8 William Harvey Research Institute, Barts and The London School of Medicine and Dentistry, Queen Mary University of London, John Vane Science Centre, Charterhouse Square, London, UK; 9 Department of Psychiatry, University of North Carolina at Chapel Hill, Chapel Hill, NC USA; 10 Wellcome Trust Centre for Human Genetics (WTCHG), University of Oxford, Oxford, UK; 11 Oxford Centre for Diabetes, Endocrinology and Metabolism (OCDEM), Oxford, UK; 12 Department of Psychology, Michigan State University, East Lansing, MI USA; 13 Section of Eating Disorders, Institute of Psychiatry, King’s College London, London, UK; 15 Department of Child and Adolescent Psychiatry, Psychosomatics and Psychotherapy, Universitätsklinikum Essen, University of Duisburg-Essen, Essen, Germany; 16 INSERM U894, Centre of Psychiatry and Neuroscience, Paris, France; 17 Sainte-Anne Hospital (CMME), University of Paris-Descartes, Paris, France; 18 Brain Center Rudolf Magnus, Department of Translational Neuroscience, University Medical Center Utrecht, Utrecht, The Netherlands; 19 Altrecht Eating Disorders Rintveld, Zeist, The Netherlands; 20 Department of Neurosciences, University of Padova, Padova, Italy; 21 Department of Psychiatry and CIBERON, University Hospital of Bellvitge-IDIBELL, Barcelona, Spain; 22 Department of Clinical Sciences, School of Medicine, University of Barcelona, Barcelona, Spain; 23 Genomics and Disease Group, Centre for Genomic Regulation (CRG), Barcelona, Spain; 24 Universitat Pompeu Fabra (UPF), Barcelona, Spain; 25 Centro de Investigación Biomédica en Red en Epidemiología y Salud Pública (CIBERESP), Barcelona, Spain; 26 Hospital del Mar Medical Research Institute (IMIM), Barcelona, Spain; 27 Department of Child and Adolescent Psychiatry, Institute of Psychiatry and Neurology, Warsaw, Poland; 28 Department of Child and Adolescent Psychiatry, Department of Psychiatry, Poznan University of Medical Sciences, Poznan, Poland; 29 Hjelt Institute, University of Helsinki, Helsinki, Finland; 30 Institute of Molecular Medicine, University of Helsinki, Helsinki, Finland; 31 Department of Mental Health and Substance Abuse Services, National Institute for Health and Welfare, Helsinki, Finland; 32 Department of Adolescent Psychiatry, Helsinki University Central Hospital, Helsinki, Finland; 33 Center for Eating Disorders Ursula, Leiden, The Netherlands; 34 Leiden University Medical Centre, Department of Psychiatry, Leiden, The Netherlands; 35 Leiden University Medical Centre, Molecular Epidemiology Section (Department of Medical Statistics), Leiden, The Netherlands; 36 Department of Psychiatry, McLean Hospital/Harvard Medical School, Belmont, MA USA; 37 Department of Genetics, Environment and Mental Health, Norwegian Institute of Public Health, Oslo, Norway; 38 Institute of Clinical Medicine, University of Oslo, Oslo, Norway; 39 Department of Psychiatry, University of Naples SUN, Naples, Italy; 40 Chair of Psychiatry, University of Salerno, Salerno, Italy; 41 Centre for Addiction and Mental Health, University of Toronto, Toronto, Canada; 42 Department of Psychiatry, University of Toronto, Toronto, Canada; 43 Eating Disorders Unit, Department of Child and Adolescent Psychiatry, Medical University of Vienna, Vienna, Austria; 44 Department of Psychiatry, University of Pennsylvania, Philadelphia, PA USA; 45 Department of Molecular and Experimental Medicine and The Scripps Translational Science Institute, The Scripps Research Institute, La Jolla, CA USA; 46 Department of Psychosomatic Research, National Institute of Mental Health, NCNP, Tokyo, Japan; 47 Department of Molecular Life Sciences, Tokai University School of Medicine, Kanagawa, Japan; 48 Estonian Genome Center, University of Tartu, Tartu, Estonia; 49 Institute of Molecular and Cell Biology, University of Tartu, Tartu, Estonia; 50 Center for Integrative Genomics, University of Lausanne, Lausanne, Switzerland; 51 Seattle University College of Nursing, Seattle, WA USA; 52 Kartini Clinic, Portland, OR USA; 53 Centre de Psychiatrie et Neurosciences – Inserm U894, Paris, France; 54 Department of Genetics, The University of North Carolina at Chapel Hill, Chapel Hill, NC USA; 55 Social, Genetic and Developmental Psychiatry Centre, Institute of Psychiatry, King’s College London, London, UK; 56 UCL Genetics Institute, Department of Genetics, Evolution and Environment, University College London, London, UK; 57 Department of Child and Adolescent Psychiatry, Psychosomatics and Psychotherapy, University Clinics RWTH Aachen, Aachen, Germany; 58 Department of Child and Adolescent Psychiatry, Psychosomatics and Psychotherapy, Charité, Berlin, Germany; 59 Department of Psychosomatic Medicine and Psychotherapy, Hannover Medical School, Hannover, Germany; 60 Department of Psychosomatic Medicine and Psychotherapy, University of Erlangen-Nuremberg, Erlangen, Germany; 61 Department of Child and Adolescent Psychiatry, Psychosomatics and Psychotherapy, University Würzburg, Würzburg, Germany; 62 Department of Child and Adolescent Psychiatry, University Hospital Carl Gustav Carus, Dresden University of Technology, Dresden, Germany; 63 Massachusetts General Hospital/Harvard Medical School, Athinoula A. Martinos Center for Biomedical Imaging, Psychiatric Neuroimaging Research Program, Charlestown, MA USA; 64 Department of Child and Adolescent Psychiatry, Psychosomatics and Psychotherapy, University Clinics RWTH Aachen, Aachen, Germany; 65 Departments of Psychosocial and Internal Medicine, Heidelberg University, Heidelberg, Germany; 66 Parklandklinik, Bad Wildungen, Germany; 67 Institute for Medical Informatics, Biometry and Epidemiology, Universitätsklinikum Essen, University of Duisburg-Essen, Essen, Germany; 68 Department of Internal Medicine VI, Psychosomatic Medicine and Psychotherapy, University Medical Hospital Tübingen, Tübingen, Germany; 69 Department of Medical Genetics, University Medical Center Utrecht, Utrecht, The Netherlands; 70 Center for Neurobehavioral Genetics, University of California, Los Angeles, Los Angeles, CA USA; 71 Brain Center Rudolf Magnus, Department of Psychiatry, University Medical Center Utrecht, Utrecht, The Netherlands; 72 Department of Social Sciences, Utrecht University, Utrecht, The Netherlands; 73 Clinical Genetics Unit, Department of Woman and Child Health, University of Padova, Padova, Italy; 74 M. Sklodowska-Curie Cancer Center and Institute of Oncology, Warsaw, Poland; 75 Department of Epidemiology, Institute of Occupational Medicine, Department of Epidemiology, Lodz, Poland; 76 Department of Clinical Nutrition, Institute of Public Health and Clinical Nutrition, University of Eastern Finland, Kuopio, Finland; 77 Netherlands Consortium for Healthy Ageing, Leiden University Medical Center, Leiden, The Netherlands; 78 Department of Nutrition and Dietetics, Harokopio University, Athens, Greece; 79 Department of Psychiatry, Athens University Medical School, Athens, Greece; 80 Eating Disorders Unit, 1st Department of Psychiatry, Athens University Medical School, Athens, Greece; 81 Adolescent Health Unit (AHU), 2nd Department of Pediatrics – Medical School, University of Athens ‘P & A Kyriakou’ Children’s Hospital, Athens, Greece; 82 Department of Psychiatry, 1st Faculty of Medicine, Charles University, Prague, Czech Republic; 83 Department of Pediatrics, 1st Faculty of Medicine, Charles University, Prague, Czech Republic; 84 Department of Medical Epidemiology and Biostatistics, Karolinska Institutet, Stockholm, Sweden; 85 Institute of Human Genetics, Department of Genomics, Life & Brain Center, University of Bonn, Bonn, Germany; 86 Institute of Neuroscience and Medicine (INM-1), Research Center Jülich, Jülich, Julich, Germany; 87 Division of Medical Genetics, Department of Biomedicine, University of Basel, Basel, Switzerland; 88 Martin-Luther-Universität Halle-Wittenberg, Klinikum der Medizinischen Fakultät, Halle/Saale, Germany; 89 Institute of Clinical Molecular Biology, University of Kiel, Kiel, Germany; 90 Institute of Epidemiology, Helmholtz Zentrum München, German Research Center for Environmental Health, Neuherberg, Germany; 91 Institute of Medical Informatics, Biometry and Epidemiology, Ludwig-Maximilians-University, Munich, Germany; 92 CNRS 8090-Institute of Biology, Pasteur Institute, Lille, France; 93 McGill University and Genome Quebec Innovation Centre, Montreal, QC Canada; 94 Division of Nephrology, Department of Internal Medicine and Medical Specialties, Columbus-Gemelly Hospitals, Catholic University, Rome, Italy; 95 Unitat de Recerca de Reumatologia (URR), Institut de Recerca Hospital Universitari Vall d’Hebron, Barcelona, Spain; 96 Genetic Epidemiology Group, International Agency for Research on Cancer (IARC), Lyon, France; 97 Virginia Institute for Psychiatric and Behavioral Genetics, Department of Psychiatry, Virginia Commonwealth University, Virginia, VA USA; 98 The Finnish Institute of Molecular Medicine Finland (FIMM), University of Helsinki, Helsinki, Finland; 99 The Program for Human and Population Genetics, The Broad Institute of MIT and Harvard, Cambridge, MA USA; 100 Finnish Institute of Occupational Health, Province of Southern Finland, Helsinki, Finland; 101 NORMENT, KG Jebsen Centre for Psychosis Research, Division of Mental Health and Addiction, Oslo University Hospital & Institute of Clinical Medicine, University of Oslo, Oslo, Norway; 102 Department of Psychology, University of Oslo, Oslo, Norway; 103 Department of Biological and Medical Psychology, University of Bergen, Bergen, Norway; 104 Kavli Research Centre for Aging and Dementia, Haraldsplass Deaconess Hospital, Bergen, Norway; 105 KG Jebsen Centre for Research on Neuropsychiatric Disorders, University of Bergen, Bergen, Norway; 106 KG Jebsen Centre for Psychosis Research, Norwegian Centre For Mental Disorders Research (NORMENT), Department of Clinical Science, University of Bergen, Bergen, Norway; 107 Dr Einar Martens Research Group for Biological Psychiatry, Center for Medical Genetics and Molecular Medicine, Haukeland University Hospital, Bergen, Norway; 108 Institute of Hygiene and Epidemiology, 1st Faculty of Medicine, Charles University, Prague, Czech Republic; 109 Department of Cancer Epidemiology and Genetics, Masaryk Memorial Cancer Institute, Brno, Czech Republic; 110 Palacky University, Olomouc, Czech Republic; 111 University Health Network and Mount Sinai Hospital, Toronto General Hospital, and Samuel Lunenfeld Research Institute, Toronto, ON Canada; 112 Departments of Psychiatry, and Genetics and Genomic Sciences, Seaver Autism Center, and the Mindich Child Health and Development Institute, Mount Sinai School of Medicine, New York, NY USA; 113 The Centre for Applied Genomics and Program in Genetics and Genome Biology, The Hospital for Sick Children, Toronto, ON Canada; 114 Department of Psychiatry and Psychotherapy, Medical University Vienna, Vienna, Austria; 115 The Institute of Environmental Medicine, Karolinska Institutet, Stockholm, Sweden; 116 Rheumatology Unit, Department of Medicine at the Karolinska University Hospital, Solna, Sweden; 117 Department of Nutrition, The University of North Carolina at Chapel Hill, Chapel Hill, NC USA; 118 Department of Psychiatry, University of Maryland School of Medicine, Baltimore, MD USA; 119 Department of Psychiatry, University of Minnesota, Minneapolis, MN USA; 120 Roseneck Hospital for Behavioral Medicine, Prien, Germany; 121 Department of Psychiatry, University of Munich (LMU), Munich, Germany; 122 New York Presbyterian Hospital, Westchester Division, Weill Medical College of Cornell University, White Plains, NY USA; 123 Laureate Psychiatric Clinic and Hospital, Tulsa, OK USA; 124 Center for Addiction and Mental Health, Toronto, Canada; 125 Department of Psychiatry, Toronto General Hospital, University Health Network, Toronto, Canada; 126 Neuropsychiatric Research Institute, Fargo, ND USA; 127 Department of Clinical Neuroscience, University of North Dakota School of Medicine and Health Sciences, Grand Forks, ND USA; 128 Department of Psychiatry and Biobehavioral Sciences, David Geffen School of Medicine, University of California at Los Angeles, Los Angeles, CA USA; 129 Neuropsychiatric Research Biotechnologies, University of Pisa, Pisa, Italy; 130 Eating Disorders Section, Institute of Psychiatry, King’s College, University of London, London, England; 131 Department of Psychology, Florida State University, Tallahassee, FL USA; 132 Department of Psychology, Georgia State University, Atlanta, GA USA; 133 Center for Health Sciences, SRI International, Menlo Park, CA USA; 134 Department of Psychiatry, University of Pennsylvania, Philadelphia, PA USA; 135 Department of Psychiatry, University of California at San Diego, San Diego, CA USA; 136 Department of Psychiatry, Brain Mind Institute EPFL—Lausanne, Center for Psychiatric Neuroscience, University of Lausanne Medical School, Lausanne, Switzerland

## Abstract

We conducted a genome-wide association study (GWAS) of anorexia nervosa (AN) using a stringently defined phenotype. Analysis of phenotypic variability led to the identification of a specific genetic risk factor that approached genome-wide significance (rs929626 in *EBF1* (Early B-Cell Factor 1); *P* = 2.04 × 10^−7^; OR = 0.7; 95% confidence interval (CI) = 0.61–0.8) with independent replication (*P* = 0.04), suggesting a variant-mediated dysregulation of leptin signaling may play a role in AN. Multiple SNPs in LD with the variant support the nominal association. This demonstrates that although the clinical and etiologic heterogeneity of AN is universally recognized, further careful sub-typing of cases may provide more precise genomic signals. In this study, through a refinement of the phenotype spectrum of AN, we present a replicable GWAS signal that is nominally associated with AN, highlighting a potentially important candidate locus for further investigation.

## Introduction

Anorexia nervosa (AN) is a complex and often chronic eating disorder characterized by inability to maintain a normal healthy body weight and a persistent fear of weight gain, resulting in extreme emaciation and even death in some cases^1^. Previous genetic and epidemiological studies have indicated a multifactorial etiology, where both genetic and environmental factors contribute to disease risk^2–7^.

As sample sizes have increased, genome-wide association studies (GWASs) of AN have begun to identify risk variants^8–10^. To further elucidate the genetic architecture of AN, we performed a GWAS using data from our previously published study^8^ consisting of 1,033 AN cases by excluding 212 patients with AN who experienced diagnostic crossover during the course of their illness. Specifically, we excluded patients who migrated from or to binge-eating disorder (BED) or bulimia nervosa (BN) as assessed with the Structured Interview for Anorexic and Bulimic Disorders^11^). Although a previous study indicated women with BN were rarely to cross over to AN^12^, we observed ~43% of AN/BN crossover cases falls into this category in our cohort, suggestive of a confounding factor. We hypothesized that this reduction in phenotypic heterogeneity, despite the fact that AN and BN may share some genetic risk factors^13^, would enhance gene discovery.

## Results

Our discovery cohort included a total of 692 female AN cases of non-Hispanic European (NHE) descent. Cases were included if they were diagnosed with restricting type and binge eating/purging type of AN as defined by DSM-IV. Both types are characterized by below-normal weight and restricted food intake. Individuals diagnosed as restricting type do not experience binge-eating episodes and do not engage in purging, such as vomiting or use of laxatives. Standard quality controls measures were applied, specifically, excluding potential cryptic relatedness and checking for population stratification (details described elsewhere^8^). The average age of onset of the case subjects was 16.3 years with a standard deviation (SD) of 3 years (Interquartile Range; IQR = 16(14–18)). The control group included 3,570 female matched healthy adolescents of NHE ancestry that had an average age of 18.3 years at the time of data analysis (SD = 5.7; IQR = 19(13–23)) (Supplementary Table 1). Associations were assessed with 507,999 SNPs genotyped on either Illumina HumanHap550 or Human610-Quad BeadChips in an additive model using logistic regression analyses with principal components adjustment, based on the principal component analysis of cases and controls (Supplementary Figure 1), resulting in significantly low level of genomic control inflation factor of 1.03 (Supplementary Figure 2). The analysis yielded one SNP (rs929626) with a *P* value of 2.04 × 10^−7^ and 4 other SNPs with marginally larger *P* values that are in strong linkage disequilibrium (*r* ^2^ > 0.8); these SNPs were selected for further analysis (Supplementary Figure 3; Supplementary Table 2).

**Figure 1:**
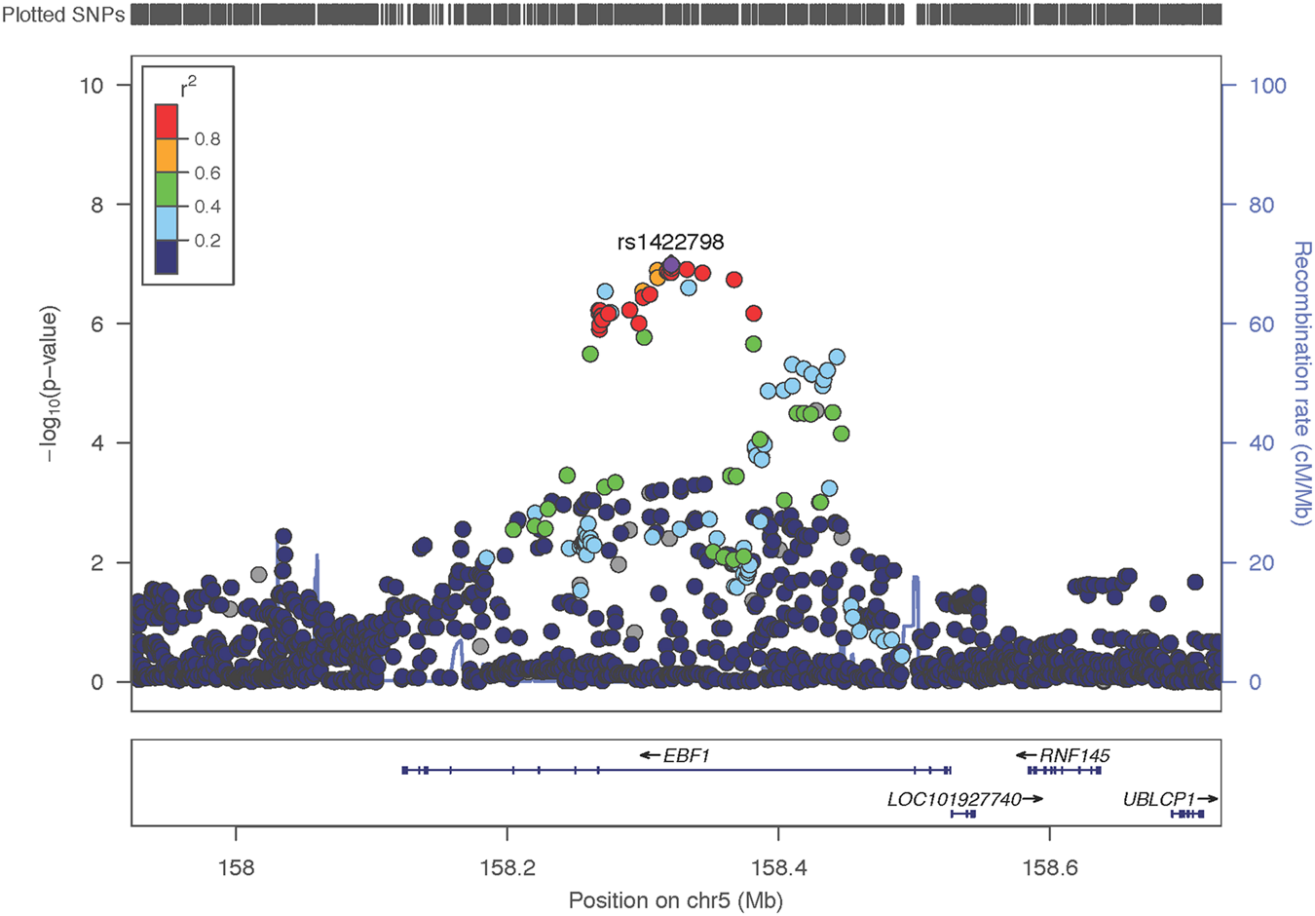
Region of genome-wide nominal association at 5q33.3. Regional plot of the *EBF1*-associated interval for the imputation analysis. Foreground shows scatter plot of the −log_10_ *P* values plotted against physical position of human reference hg19. Background shows estimated recombination rates plotted to reflect the local LD structure. The color of the dots represents the strength of LD between the top SNP (rs929626) and its proxies (red, *r* ^2^ ≥ 0.8; orange, 0.8 > *r* ^2^ ≥ 0.6; green, 0.6 > *r* ^2^ ≥ 0.4; blue and navy, *r* ^2^ < 0.4). Genes, position of exons, and direction of transcription from UCSC genome browser (http://genome.ucsc.edu) are noted.

Using imputation analysis based on data from the 1000 Genomes Project (Phase I integrated variant set, v2, March 2012), we subsequently tested associations with SNPs (imputed info > 0.5, minor allele frequency (MAF) > 0.05) located in a 200-kb window centered on the SNP rs929626. We observed association with a series of markers around this region, of which 34 SNPs supported suggestive associations (*P* < 1.0 × 10^−6^) with both imputed and genotyped SNPs, which were in high LD with AN (Fig. 1; Supplementary Table 3). This suggests that the single markers demonstrating nominal association in the GWAS are likely to be true positives.

We further explored this finding using the meta-analysis results from 15 previously reported AN cohorts^10^. Interestingly, two SNPs were also nominally significant (rs929626 with *P* = 0.037 and rs17543752 with *P* = 0.05) in the same direction as in the GWAS (Table 1 **)**. Meta-analysis results in a P value of 1.52 × 10^−7^.

**Table 1:** Association results for the lead genotyped SNP.

SNP	Study	MA	OR	SE	L95	U95	P
rs929626	CHOP	C	0.7004	0.06855	0.6123	0.8011	2.04E-07
	PGC-ED	C	0.938252	0.027953	0.883465	0.996437	0.037887

Abbreviations: MA, minor allele; OR, odds ratio; SE, standard error; L95, lower 95% confidence interval; U95, upper 95% confidence interval; P, P-value.

We next used the ENCODE project^14^ data to predict possible functional effect of the SNPs identified in this study. The top SNP, rs929626, and other significant markers located in the 6th intron of the *EBF1* gene (Early B-Cell Factor 1), as well as two SNPs (rs113252656 and rs1081071) flanking the top SNP rs929626 at *r* ^2^ > 0.5 function as binding sites for EBF1 itself (HaploReg v4.1; ref. 15). This suggests that these genetic variants may modulate the expression of *EBF1*. Indeed, we observed a positive correlation with the rs929626 C allele carriers compared with TT homozygotes on the *EBF1* expression level in nine independent subjects (the FPKM value for TT homozygotes (3 subjects) versus C allele carriers (6 individuals) is 5.0 versus 6.4) with both whole genome sequencing data of blood and corresponding RNA-Seq data of heart right ventricle selected from the Pediatric Cardiac Genomics Consortium cohort (dbGaP Study Accession: phs000571.v3.p2). By using the Genotype-Tissue Expression Portal database (http://www.gtexportal.org), we also observed nominally significant expression quantitative trait loci (eQTLs) association (*P* = 0.0024, tested in 97 samples) in the putamen for rs929626 in the same direction. A few comorbid psychiatric disorders have been linked with the function of the putamen, such as anxiety, obsessive-compulsive disorder and attention deficit-hyperactivity disorder^16–18^. Taken together, these suggest the minor allele C carriers have relatively higher *EBF1* expression.

## Discussion

*EBF1* encodes a transcription factor that originally thought to function as necessary for the development of the immune system^19^, but it has since been shown to regulate the development of both osteoblast and adipocyte lineages^20–22^. Two *EBF1* variants, rs11953630-T and rs9313772-T, showed significant association at genome-wide level (*P* < 5 × 10^−10^) in a study testing blood pressure in European whites^23, 24^. In addition, rs17056278-C was also identified as a metabolic risk allele, interacting with psychosocial stress to contribute to increased hip circumference (*P* = 3 × 10^−8^)^25^. However none of these is in LD with any markers in our identified locus. In animal studies, *Ebf1*−/− mice showed increased adipose tissue within marrow, whereas peripheral white adipose tissue was severely reduced. Circulating levels of leptin, a hormone released by adipocytes and one of the major players in food intake regulation, were also decreased in *Ebf1*−/− mice compared with controls^26^. This concurs with the reported generalized loss of accumulation of subcutaneous and visceral adipose accompanied by significant increases in yellow marrow in AN patients^27, 28^. Also notable is the finding that circulating levels of leptin are very low in AN patients^29, 30^ and a decline in levels of circulating leptin can lead to changes in brain activity in areas involved in regulatory, emotional, and cognitive control of appetite^5^.

Understanding the genetics of AN is currently a major within-field initiative, in parallel to other neuropsychiatric/neurodevelopmental disorders such as schizophrenia, bipolar disorder, and autism spectrum disorders. Although the clinical and etiologic heterogeneity is universally recognized, in practice, many studies still failed to account for sample heterogeneity. In this study, by focusing on individuals with AN who have not crossed over to BN or BED, we have identified a marginally replicating GWAS signal that approached genome-wide significance. One limitation of our study is that all participants may not yet have experienced the full course of their eating disorder (The average duration of follow-up was 8.6 years with a SD of 7.0 years in the discovery cohort, while the average crossover time was 2.8 years with a SD of 2.6 years for the excluded AN patients), and a portion of the sample may develop BN or BED at later stages of illness. This would represent a conservative bias and underscores the importance of further investigation of this locus in the future focusing on individuals with lifetime AN who have never crossed over to other eating disorder presentations.

## Methods

### Discovery data set and quality control

We conducted a GWAS using data from our previously published study^8^ consisting of 1,033 AN cases by excluding 212 patients with AN who experienced diagnostic crossover during the course of their illness (i.e. migrated from or to binge-eating disorder (BED) or bulimia nervosa (BN) as assessed with the Structured Interview for Anorexic and Bulimic Disorders^11^) plus 100 patients without such information. A total of 692 female AN cases and 3,570 female matched controls that were carefully selected from Center for Applied Genomics (CAG) database were included in the analysis after Standard quality controls, namely, excluding potential cryptic relatedness and checking for population stratification by using the PLINK software^31^ version 1.90a. The Research Ethics Board of CHOP and other participating centers approved the study. Informed consent was obtained from all adult participants and from a parent or legal guardian in the case of children and all work followed was in accordance with an IRB-approved protocol.

### Association tests

For the genome-wide association analysis for SNPs, we utilized the PLINK software^31^ version 1.90a, through Cochran–Armitage trend test.

### Expression studies

The extended locus around associated SNP was then defined by identification of all SNPs showing *r* ^*2*^ > 0.5. Linkage disequilibrium (LD) was defined with the HaploReg v4.1 (ref. 15) based on Phase I of the 1000 Genomes project. Variants showing evidence of LD with associated AN variants were explored for impact on gene function via regulatory function (including eQTLs) by HaploReg v4.1, which both collate data from the Encyclopedia of DNA Elements (ENCODE)^14^. We also referred to the Genotype-Tissue Expression Portal database (http://www.gtexportal.org) for eQTLs analysis.

## Electronic supplementary material

Supplementary Appendix
