# Erratum: A genome-wide association study of anorexia nervosa suggests a risk locus implicated in dysregulated leptin signaling

**DOI:** 10.1038/s41598-017-06409-3

**Published:** 2017-08-21

**Authors:** Dong Li, Xiao Chang, John J. Connolly, Lifeng Tian, Yichuan Liu, Elizabeth J. Bhoj, Nora Robinson, Debra Abrams, Yun R. Li, Jonathan P. Bradfield, Cecilia E. Kim, Jin Li, Fengxiang Wang, James Snyder, Maria Lemma, Cuiping Hou, Zhi Wei, Yiran Guo, Haijun Qiu, Frank D. Mentch, Kelly A. Thomas, Rosetta M. Chiavacci, Roger Cone, Bingshan Li, Patrick A. Sleiman, Eating Disorders Working Group of the Psychiatric Genomics Consortium, Vesna Boraska Perica, Christopher S. Franklin, James A. B. Floyd, Laura M. Thornton, Laura M. Huckins, Lorraine Southam, N. William Rayner, Ioanna Tachmazidou, Kelly L. Klump, Janet Treasure, Ulrike Schmidt, Federica Tozzi, Kirsty Kiezebrink, Johannes Hebebrand, Philip Gorwood, Roger A. H. Adan, Martien J. H. Kas, Angela Favaro, Paolo Santonastaso, Fernando Fernánde-Aranda, Monica Gratacos, Filip Rybakowski, Monika Dmitrzak-Weglarz, Jaakko Kaprio, Anna Keski-Rahkonen, Anu Raevuori-Helkamaa, Eric F. Van Furth, Margarita C. T. Slof-Op’t Landt, James I. Hudson, Ted Reichborn-Kjennerud, Gun Peggy S. Knudsen, Palmiero Monteleone, Allan S. Kaplan, Andreas Karwautz, Wade H. Berrettini, Nicholas J. Schork, Tetsuya Ando, Hidetoshi Inoko, Tõnu Esko, Krista Fischer, Katrin Männik, Andres Metspalu, Jessica H. Baker, Janiece E. DeSocio, Christopher E. Hilliard, Julie K. O’Toole, Jacques Pantel, Jin P. Szatkiewicz, Stephanie Zerwas, Oliver S. P. Davis, Sietske Helder, Katharina Bühren, Roland Burghardt, Martina de Zwaan, Karin Egberts, Stefan Ehrlich, Beate Herpertz-Dahlmann, Wolfgang Herzog, Hartmut Imgart, André Scherag, Stephan Zipfel, Claudette Boni, Nicolas Ramoz, Audrey Versini, Unna N. Danner, Judith Hendriks, Bobby P. C. Koeleman, Roel A. Ophoff, Eric Strengman, Annemarie A. van Elburg, Alice Bruson, Maurizio Clementi, Daniela Degortes, Monica Forzan, Elena Tenconi, Elisa Docampo, Geòrgia Escaramís, Susana Jiménez-Murcia, Jolanta Lissowska, Andrzej Rajewski, Neonila Szeszenia-Dabrowska, Agnieszka Slopien, Joanna Hauser, Leila Karhunen, Ingrid Meulenbelt, P. Eline Slagboom, Alfonso Tortorella, Mario Maj, George Dedoussis, Dimitris Dikeos, Fragiskos Gonidakis, Konstantinos Tziouvas, Artemis Tsitsika, Hana Papezova, Lenka Slachtova, Debora Martaskova, James L. Kennedy, Robert D. Levitan, Zeynep Yilmaz, Julia Huemer, Doris Koubek, Elisabeth Merl, Gudrun Wagner, Paul Lichtenstein, Gerome Breen, Sarah Cohen-Woods, Anne Farmer, Peter McGuffin, Sven Cichon, Ina Giegling, Stefan Herms, Dan Rujescu, Stefan Schreiber, H-Erich Wichmann, Christian Dina, Rob Sladek, Giovanni Gambaro, Nicole Soranzo, Antonio Julia, Sara Marsal, Raquel Rabionet, Valerie Gaborieau, Danielle M. Dick, Aarno Palotie, Samuli Ripatti, Elisabeth Widén, Ole A. Andreassen, Thomas Espeseth, Astri Lundervold, Ivar Reinvang, Vidar M. Steen, Stephanie Le Hellard, Morten Mattingsdal, Ioanna Ntalla, Vladimir Bencko, Lenka Foretova, Vladimir Janout, Marie Navratilova, Steven Gallinger, Dalila Pinto, Stephen W. Scherer, Harald Aschauer, Laura Carlberg, Alexandra Schosser, Lars Alfredsson, Bo Ding, Lars Klareskog, Leonid Padyukov, Chris Finan, Gursharan Kalsi, Marion Roberts, Jeff C. Barrett, Xavier Estivill, Anke Hinney, Patrick F. Sullivan, Eleftheria Zeggini, Cynthia M. Bulik, Price Foundation Collaborative Group, Harry Brandt, Steve Crawford, Scott Crow, Manfred M. Fichter, Katherine A. Halmi, Craig Johnson, Allan S. Kaplan, Maria C. La Via, James Mitchell, Michael Strober, Alessandro Rotondo, Janet Treasure, D. Blake Woodside, Cynthia M. Bulik, Pamela K. Keel, Kelly L. Klump, Lisa Lilenfeld, Laura M. Thornton, Andrew W. Bergen, Wade Berrettini, Walter Kaye, Pierre Magistretti, Hakon Hakonarson

**Affiliations:** 1 Center for Applied Genomics, Children’s Hospital of Philadelphia, Philadelphia, PA USA; 2 Department of Molecular Physiology and Biophysics, Vanderbilt University, Nashville, TN USA; 3 Department of Human Genetics, Children’s Hospital of Philadelphia, Philadelphia, PA USA; 4 Department of Pediatrics, The Perelman School of Medicine, University of Pennsylvania, Philadelphia, PA USA; 5 Present Address: Department of Molecular and Integrative Physiology, University of Michigan, Ann Arbor, MI USA; 14 Health Services Research Unit, University of Aberdeen, Aberdeen, UK; 6 Wellcome Trust Sanger Institute, Wellcome Trust Genome Campus, Hinxton, Cambridge UK; 7 University of Split School of Medicine, Split, Croatia; 8 William Harvey Research Institute, Barts and The London School of Medicine and Dentistry, Queen Mary University of London, John Vane Science Centre, Charterhouse Square, London, UK; 9 Department of Psychiatry, University of North Carolina at Chapel Hill, Chapel Hill, NC USA; 10 Wellcome Trust Centre for Human Genetics (WTCHG), University of Oxford, Oxford, UK; 11 Oxford Centre for Diabetes, Endocrinology and Metabolism (OCDEM), Oxford, UK; 12 Department of Psychology, Michigan State University, East Lansing, MI USA; 13 Section of Eating Disorders, Institute of Psychiatry, King’s College London, London, UK; 15 Department of Child and Adolescent Psychiatry, Psychosomatics and Psychotherapy, Universitätsklinikum Essen, University of Duisburg-Essen, Essen, Germany; 16 INSERM U894, Centre of Psychiatry and Neuroscience, Paris, France; 17 Sainte-Anne Hospital (CMME), University of Paris-Descartes, Paris, France; 18 Brain Center Rudolf Magnus, Department of Translational Neuroscience, University Medical Center Utrecht, Utrecht, The Netherlands; 19 Altrecht Eating Disorders Rintveld, Zeist, The Netherlands; 20 Department of Neurosciences, University of Padova, Padova, Italy; 21 Department of Psychiatry and CIBERON, University Hospital of Bellvitge-IDIBELL, Barcelona, Spain; 22 Department of Clinical Sciences, School of Medicine, University of Barcelona, Barcelona, Spain; 23 Genomics and Disease Group, Centre for Genomic Regulation (CRG), Barcelona, Spain; 24 Universitat Pompeu Fabra (UPF), Barcelona, Spain; 25 Centro de Investigación Biomédica en Red en Epidemiología y Salud Pública (CIBERESP), Barcelona, Spain; 26 Hospital del Mar Medical Research Institute (IMIM), Barcelona, Spain; 27 Department of Child and Adolescent Psychiatry, Institute of Psychiatry and Neurology, Warsaw, Poland; 28 Department of Child and Adolescent Psychiatry, Department of Psychiatry, Poznan University of Medical Sciences, Poznan, Poland; 29 Hjelt Institute, University of Helsinki, Helsinki, Finland; 30 Institute of Molecular Medicine, University of Helsinki, Helsinki, Finland; 31 Department of Mental Health and Substance Abuse Services, National Institute for Health and Welfare, Helsinki, Finland; 32 Department of Adolescent Psychiatry, Helsinki University Central Hospital, Helsinki, Finland; 33 Center for Eating Disorders Ursula, Leiden, The Netherlands; 34 Leiden University Medical Centre, Department of Psychiatry, Leiden, The Netherlands; 35 Leiden University Medical Centre, Molecular Epidemiology Section (Department of Medical Statistics), Leiden, The Netherlands; 36 Department of Psychiatry, McLean Hospital/Harvard Medical School, Belmont, MA USA; 37 Department of Genetics, Environment and Mental Health, Norwegian Institute of Public Health, Oslo, Norway; 38 Institute of Clinical Medicine, University of Oslo, Oslo, Norway; 39 Department of Psychiatry, University of Naples SUN, Naples, Italy; 40 Chair of Psychiatry, University of Salerno, Salerno, Italy; 41 Centre for Addiction and Mental Health, University of Toronto, Toronto, Canada; 42 Department of Psychiatry, University of Toronto, Toronto, Canada; 43 Eating Disorders Unit, Department of Child and Adolescent Psychiatry, Medical University of Vienna, Vienna, Austria; 44 Department of Psychiatry, University of Pennsylvania, Philadelphia, PA USA; 45 Department of Molecular and Experimental Medicine and The Scripps Translational Science Institute, The Scripps Research Institute, La Jolla, CA USA; 46 Department of Psychosomatic Research, National Institute of Mental Health, NCNP, Tokyo, Japan; 47 Department of Molecular Life Sciences, Tokai University School of Medicine, Kanagawa, Japan; 48 Estonian Genome Center, University of Tartu, Tartu, Estonia; 49 Institute of Molecular and Cell Biology, University of Tartu, Tartu, Estonia; 50 Center for Integrative Genomics, University of Lausanne, Lausanne, Switzerland; 51 Seattle University College of Nursing, Seattle, WA USA; 52 Kartini Clinic, Portland, OR USA; 53 Centre de Psychiatrie et Neurosciences – Inserm U894, Paris, France; 54 Department of Genetics, The University of North Carolina at Chapel Hill, Chapel Hill, NC USA; 55 Social, Genetic and Developmental Psychiatry Centre, Institute of Psychiatry, King’s College London, London, UK; 56 UCL Genetics Institute, Department of Genetics, Evolution and Environment, University College London, London, UK; 57 Department of Child and Adolescent Psychiatry, Psychosomatics and Psychotherapy, University Clinics RWTH Aachen, Aachen, Germany; 58 Department of Child and Adolescent Psychiatry, Psychosomatics and Psychotherapy, Charité, Berlin, Germany; 59 Department of Psychosomatic Medicine and Psychotherapy, Hannover Medical School, Hannover, Germany; 60 Department of Psychosomatic Medicine and Psychotherapy, University of Erlangen-Nuremberg, Erlangen, Germany; 61 Department of Child and Adolescent Psychiatry, Psychosomatics and Psychotherapy, University Würzburg, Würzburg, Germany; 62 Department of Child and Adolescent Psychiatry, University Hospital Carl Gustav Carus, Dresden University of Technology, Dresden, Germany; 63 Massachusetts General Hospital/Harvard Medical School, Athinoula A. Martinos Center for Biomedical Imaging, Psychiatric Neuroimaging Research Program, Charlestown, MA USA; 64 Department of Child and Adolescent Psychiatry, Psychosomatics and Psychotherapy, University Clinics RWTH Aachen, Aachen, Germany; 65 Departments of Psychosocial and Internal Medicine, Heidelberg University, Heidelberg, Germany; 66 Parklandklinik, Bad Wildungen, Germany; 67 Institute for Medical Informatics, Biometry and Epidemiology, Universitätsklinikum Essen, University of Duisburg-Essen, Essen, Germany; 68 Department of Internal Medicine VI, Psychosomatic Medicine and Psychotherapy, University Medical Hospital Tübingen, Tübingen, Germany; 69 Department of Medical Genetics, University Medical Center Utrecht, Utrecht, The Netherlands; 70 Center for Neurobehavioral Genetics, University of California, Los Angeles, Los Angeles, CA USA; 71 Brain Center Rudolf Magnus, Department of Psychiatry, University Medical Center Utrecht, Utrecht, The Netherlands; 72 Department of Social Sciences, Utrecht University, Utrecht, The Netherlands; 73 Clinical Genetics Unit, Department of Woman and Child Health, University of Padova, Padova, Italy; 74 M. Sklodowska-Curie Cancer Center and Institute of Oncology, Warsaw, Poland; 75 Department of Epidemiology, Institute of Occupational Medicine, Department of Epidemiology, Lodz, Poland; 76 Department of Clinical Nutrition, Institute of Public Health and Clinical Nutrition, University of Eastern Finland, Kuopio, Finland; 77 Netherlands Consortium for Healthy Ageing, Leiden University Medical Center, Leiden, The Netherlands; 78 Department of Nutrition and Dietetics, Harokopio University, Athens, Greece; 79 Department of Psychiatry, Athens University Medical School, Athens, Greece; 80 Eating Disorders Unit, 1st Department of Psychiatry, Athens University Medical School, Athens, Greece; 81 Adolescent Health Unit (AHU), 2nd Department of Pediatrics – Medical School, University of Athens ‘P & A Kyriakou’ Children’s Hospital, Athens, Greece; 82 Department of Psychiatry, 1st Faculty of Medicine, Charles University, Prague, Czech Republic; 83 Department of Pediatrics, 1st Faculty of Medicine, Charles University, Prague, Czech Republic; 84 Department of Medical Epidemiology and Biostatistics, Karolinska Institutet, Stockholm, Sweden; 85 Institute of Human Genetics, Department of Genomics, Life & Brain Center, University of Bonn, Bonn, Germany; 86 Institute of Neuroscience and Medicine (INM-1), Research Center Jülich, Jülich, Julich, Germany; 87 Division of Medical Genetics, Department of Biomedicine, University of Basel, Basel, Switzerland; 88 Martin-Luther-Universität Halle-Wittenberg, Klinikum der Medizinischen Fakultät, Halle/Saale, Germany; 89 Institute of Clinical Molecular Biology, University of Kiel, Kiel, Germany; 90 Institute of Epidemiology, Helmholtz Zentrum München, German Research Center for Environmental Health, Neuherberg, Germany; 91 Institute of Medical Informatics, Biometry and Epidemiology, Ludwig-Maximilians-University, Munich, Germany; 92 CNRS 8090-Institute of Biology, Pasteur Institute, Lille, France; 93 McGill University and Genome Quebec Innovation Centre, Montreal, QC Canada; 94 Division of Nephrology, Department of Internal Medicine and Medical Specialties, Columbus-Gemelly Hospitals, Catholic University, Rome, Italy; 95 Unitat de Recerca de Reumatologia (URR), Institut de Recerca Hospital Universitari Vall d’Hebron, Barcelona, Spain; 96 Genetic Epidemiology Group, International Agency for Research on Cancer (IARC), Lyon, France; 97 Virginia Institute for Psychiatric and Behavioral Genetics, Department of Psychiatry, Virginia Commonwealth University, Virginia, VA USA; 98 The Finnish Institute of Molecular Medicine Finland (FIMM), University of Helsinki, Helsinki, Finland; 99 The Program for Human and Population Genetics, The Broad Institute of MIT and Harvard, Cambridge, MA USA; 100 Finnish Institute of Occupational Health, Province of Southern Finland, Helsinki, Finland; 101 NORMENT, KG Jebsen Centre for Psychosis Research, Division of Mental Health and Addiction, Oslo University Hospital & Institute of Clinical Medicine, University of Oslo, Oslo, Norway; 102 Department of Psychology, University of Oslo, Oslo, Norway; 103 Department of Biological and Medical Psychology, University of Bergen, Bergen, Norway; 104 Kavli Research Centre for Aging and Dementia, Haraldsplass Deaconess Hospital, Bergen, Norway; 105 KG Jebsen Centre for Research on Neuropsychiatric Disorders, University of Bergen, Bergen, Norway; 106 KG Jebsen Centre for Psychosis Research, Norwegian Centre For Mental Disorders Research (NORMENT), Department of Clinical Science, University of Bergen, Bergen, Norway; 107 Dr Einar Martens Research Group for Biological Psychiatry, Center for Medical Genetics and Molecular Medicine, Haukeland University Hospital, Bergen, Norway; 108 Institute of Hygiene and Epidemiology, 1st Faculty of Medicine, Charles University, Prague, Czech Republic; 109 Department of Cancer Epidemiology and Genetics, Masaryk Memorial Cancer Institute, Brno, Czech Republic; 110 Palacky University, Olomouc, Czech Republic; 111 University Health Network and Mount Sinai Hospital, Toronto General Hospital, and Samuel Lunenfeld Research Institute, Toronto, ON Canada; 112 Departments of Psychiatry, and Genetics and Genomic Sciences, Seaver Autism Center, and the Mindich Child Health and Development Institute, Mount Sinai School of Medicine, New York, NY USA; 113 The Centre for Applied Genomics and Program in Genetics and Genome Biology, The Hospital for Sick Children, Toronto, ON Canada; 114 Department of Psychiatry and Psychotherapy, Medical University Vienna, Vienna, Austria; 115 The Institute of Environmental Medicine, Karolinska Institutet, Stockholm, Sweden; 116 Rheumatology Unit, Department of Medicine at the Karolinska University Hospital, Solna, Sweden; 117 Department of Nutrition, The University of North Carolina at Chapel Hill, Chapel Hill, NC USA; 118 Department of Psychiatry, University of Maryland School of Medicine, Baltimore, MD USA; 119 Department of Psychiatry, University of Minnesota, Minneapolis, MN USA; 120 Roseneck Hospital for Behavioral Medicine, Prien, Germany; 121 Department of Psychiatry, University of Munich (LMU), Munich, Germany; 122 New York Presbyterian Hospital, Westchester Division, Weill Medical College of Cornell University, White Plains, NY USA; 123 Laureate Psychiatric Clinic and Hospital, Tulsa, OK USA; 124 Center for Addiction and Mental Health, Toronto, Canada; 125 Department of Psychiatry, Toronto General Hospital, University Health Network, Toronto, Canada; 126 Neuropsychiatric Research Institute, Fargo, ND USA; 127 Department of Clinical Neuroscience, University of North Dakota School of Medicine and Health Sciences, Grand Forks, ND USA; 128 Department of Psychiatry and Biobehavioral Sciences, David Geffen School of Medicine, University of California at Los Angeles, Los Angeles, CA USA; 129 Neuropsychiatric Research Biotechnologies, University of Pisa, Pisa, Italy; 130 Eating Disorders Section, Institute of Psychiatry, King’s College, University of London, London, England; 131 Department of Psychology, Florida State University, Tallahassee, FL USA; 132 Department of Psychology, Georgia State University, Atlanta, GA USA; 133 Center for Health Sciences, SRI International, Menlo Park, CA USA; 134 Department of Psychiatry, University of Pennsylvania, Philadelphia, PA USA; 135 Department of Psychiatry, University of California at San Diego, San Diego, CA USA; 136 Department of Psychiatry, Brain Mind Institute EPFL—Lausanne, Center for Psychiatric Neuroscience, University of Lausanne Medical School, Lausanne, Switzerland

*Scientific Reports* **7**:3847; doi:10.1038/s41598-017-01674-8; Article published online 19 June 2017

The original HTML version of this Article contained errors. In the author list, Hakon Hakonarson was incorrectly listed before the ‘Eating Disorders Working Group of the Psychiatric Genomics Consortium’ and ‘Price Foundation Collaborative Group’.

In addition, the affiliations for the ‘Eating Disorders Working Group of the Psychiatric Genomics Consortium’ and ‘Price Foundation Collaborative Group’ were missing from the Author information section.

These errors have now been corrected in the HTML version of the Article; the PDF version was correct from the time of publication.

