# Supplementary material for: A genome-wide association study of anorexia nervosa suggests a risk locus implicated in dysregulated leptin signaling
Source: Sci Rep. 2017 Jun 19;7:3847. doi: 10.1038/s41598-017-01674-8 (PMC5476671; doi:10.1038/s41598-017-01674-8)
Supplement: Supplementary file 1 — Supplementary Appendix [file 41598_2017_1674_MOESM1_ESM.pdf]

A genome-wide association study of anorexia nervosa suggests a risk locus implicated in dysregulated leptin signaling

Dong Li[1,#,*], Xiao Chang[1,#], John J Connolly[1], Lifeng Tian[1], Yichuan Liu[1], Elizabeth J Bhoj[1], Nora Robinson[1], Debra Abrams[1], Yun R. Li[1], Jonathan P Bradfield[1], Cecilia E Kim[1], Jin Li[1], Fengxiang Wang[1], James Snyder[1], Maria Lemma[1], Cuiping Hou[1], Zhi Wei[1], Yiran Guo[1], Haijun Qiu[1], Frank D Mentch[1], Kelly A Thomas[1], Rosetta M Chiavacci[1], Roger Cone[2], Bingshan Li[2], Patrick A Sleiman[1], Eating Disorders Working Group of the Psychiatric Genomics Consortium[5], Price Foundation Collaborative Group[6], Hakon Hakonarson[1,3,4,*]

1. Center for Applied Genomics, Children's Hospital of Philadelphia, Philadelphia, PA
2. Department of Molecular Physiology and Biophysics, Vanderbilt University, Nashville, TN
3. Department of Human Genetics, Children's Hospital of Philadelphia, Philadelphia, PA
4. Department of Pediatrics, The Perelman School of Medicine, University of Pennsylvania, Philadelphia, PA
5. PGC-ED members are listed after the Acknowledgements
6. Price Foundation Collaborative Group members are listed after the Acknowledgements

Supplementary Materials

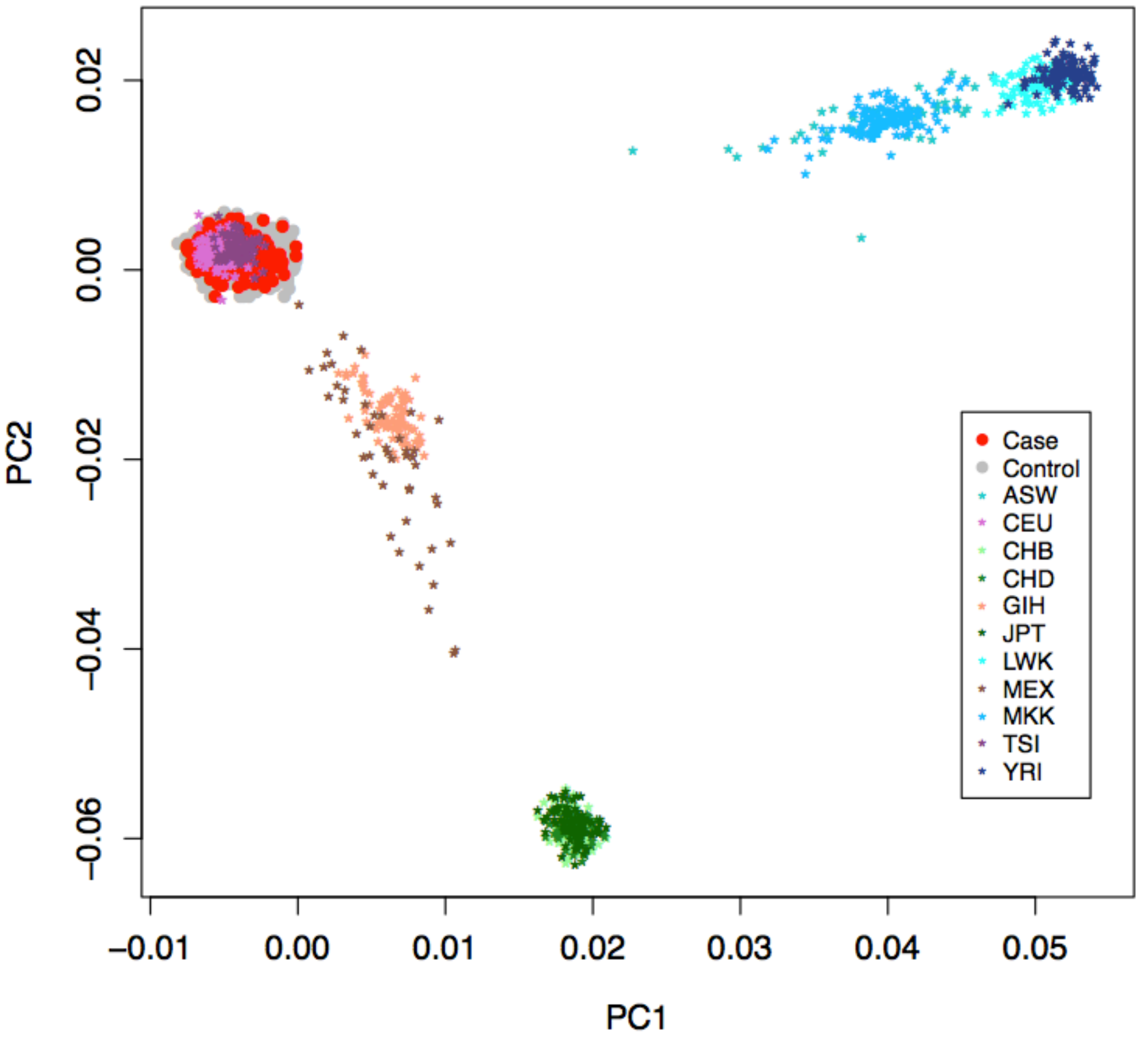

Supplementary Figure 1. Principal component analysis of all genotyped subjects (CHOP) together with 11 HapMap 3 populations (CEU, YRI, CHB, JPT, etc.).

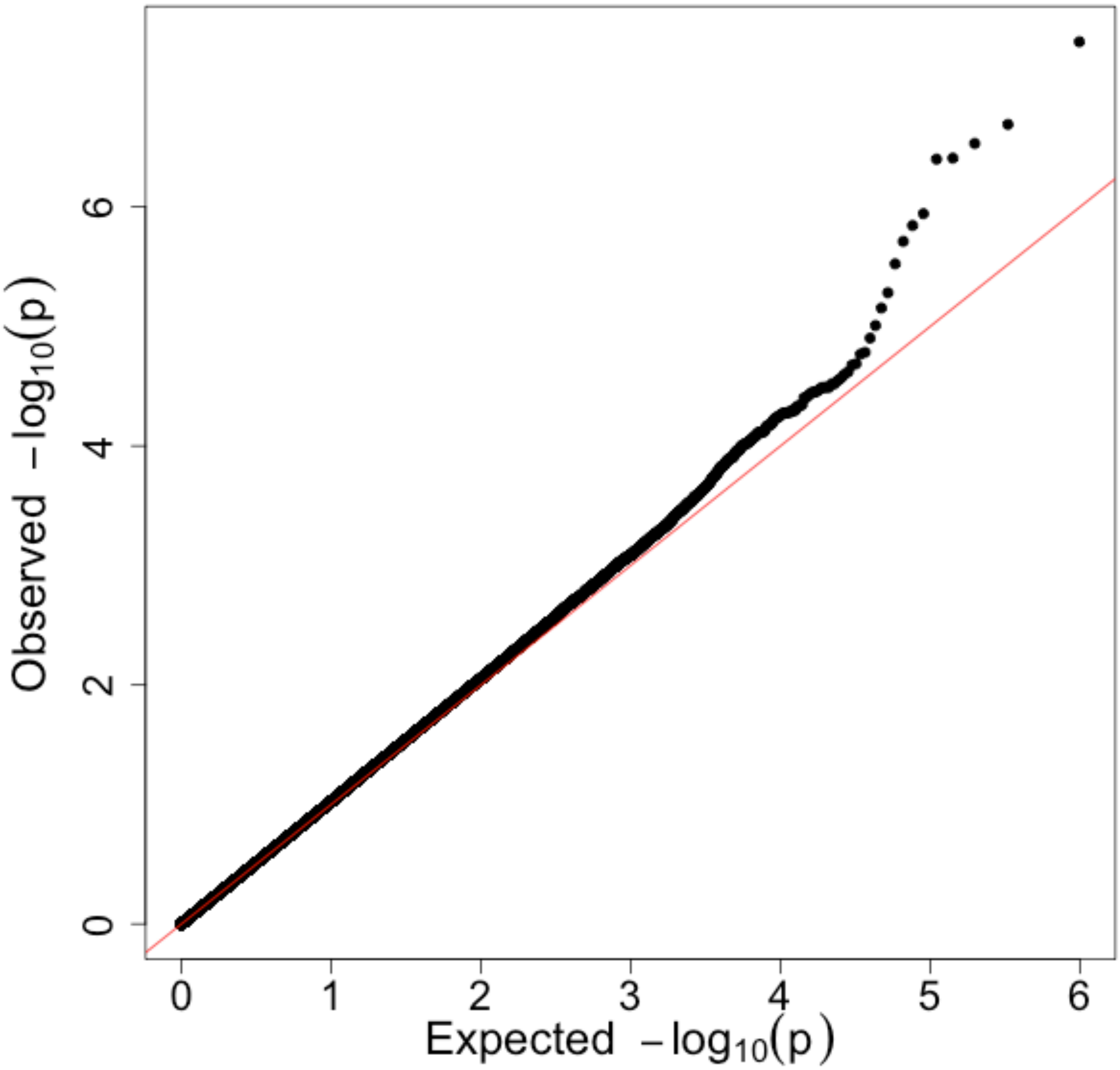

Supplementary Figure 2. The quantile-quantile plot of $-\log_{10} P$ values for genome-wide association tests on SNPs.

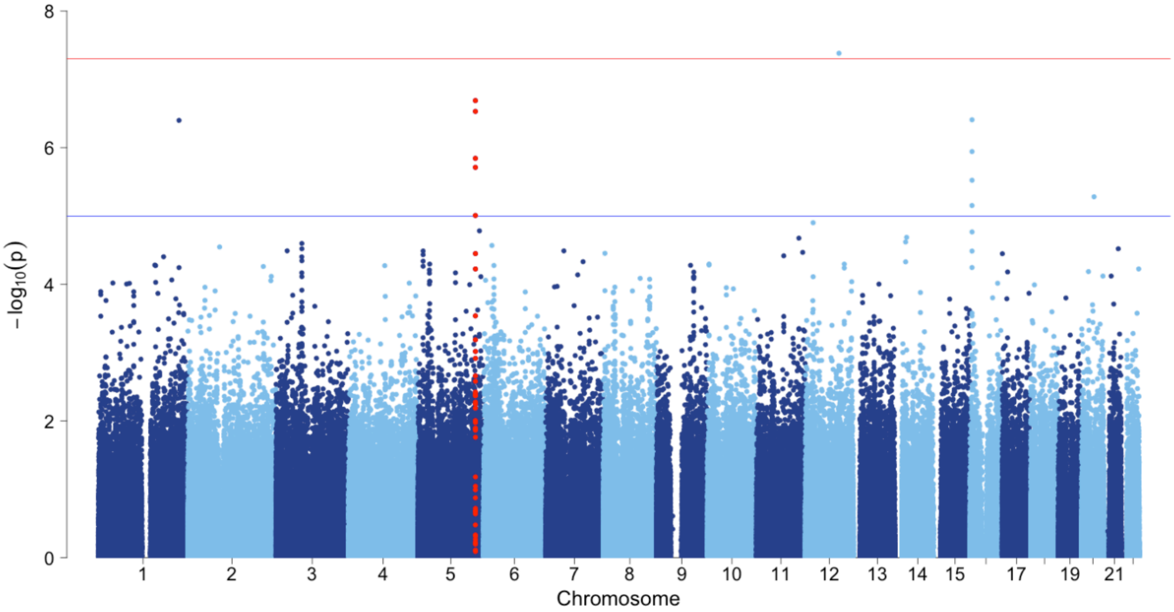

Supplementary Figure 3. The Manhattan plot of $-\log_{10} P$ values versus genomic coordinates for whole-genome SNPs

Supplementary Table 1. Summary of the cases/controls in this study.

Cohort	Age (onset age for case and assessment age for control)			Number (all females)
	Mean	SD	Median and lower to upper quartile range	
Case	16.2	3	16 (14-18)	692
Control	18.3	5.7	19 (13-23)	3570

Supplementary Table 2. Association results for the genotyped SNP ($P < 1e\text{-}5$)

Chr	Postion	Marker	MA	OR	SE	L95	U95	P
1	224794913	rs7535525	C	1.869	0.1234	1.468	2.38	3.99E-07
5	158270225	rs7707918	C	0.7115	0.07062	0.6195	0.8171	1.43E-06
5	158274071	rs1978706	T	1.36	0.06948	1.186	1.558	9.83E-06
5	158296996	rs2072495	A	0.7147	0.07057	0.6224	0.8207	1.94E-06
5	158310631	rs929626	C	0.7004	0.06855	0.6123	0.8011	2.04E-07
5	158332380	rs17543752	C	0.6927	0.0716	0.602	0.7971	2.94E-07
12	94031881	rs2196426	T	2.821	0.1891	1.947	4.086	4.15E-08
16	5794312	rs9941024	C	0.7301	0.07	0.6365	0.8375	7.02E-06
16	5802062	rs7200175	C	0.7234	0.06931	0.6315	0.8287	2.99E-06
16	5808871	rs7205418	A	0.7074	0.07112	0.6154	0.8133	1.14E-06
16	5819274	rs2043633	G	0.6958	0.07149	0.6048	0.8004	3.91E-07
20	34987833	rs6072022	T	1.37	0.06915	1.197	1.569	5.23E-06

Abbreviations: Chr, chromosome; MA, minor allele; OR, odds ratio; SE, standard error; L95, lower 95% confidence interval; U95, upper 95% confidence interval; P, *P*-value

Supplementary Table 3. Association results for the imputed and genotyped SNPs (P < 1e-5)

Chr	Postion	Marker	A	B	MAF of cases	MAF of controls	OR	SE	L95	U95	P
5	158261163	rs17715065	C	T	0.53422	0.466544	0.762527	0.0596573	0.679419	0.8558	3.21E-06
5	158267423	rs891905	T	C	0.352788	0.423768	0.741204	0.0619748	0.657498	0.835566	5.94E-07
5	158267473	rs891904	G	A	0.354273	0.424982	0.742336	0.061951	0.658571	0.836756	6.57E-07
5	158267958	rs4546365	C	T	0.351897	0.420824	0.747279	0.0619485	0.662837	0.842478	1.24E-06
5	158267974	rs4594837	A	C	0.354772	0.424097	0.746656	0.0619808	0.662421	0.841602	1.06E-06
5	158268426	rs6875710	C	T	0.356245	0.427103	0.742286	0.0620044	0.658617	0.836584	6.05E-07
5	158269081	rs10052777	T	C	0.354449	0.424859	0.743282	0.0618283	0.659417	0.837813	7.64E-07
5	158269591	rs62385360	C	T	0.353125	0.423698	0.742509	0.0618038	0.65867	0.837019	7.31E-07
5	158269734	rs17715535	G	A	0.353157	0.423708	0.742583	0.0617925	0.658737	0.837101	7.40E-07
5	158270225	rs7707918	A	C	0.354046	0.42423	0.743887	0.0617308	0.659934	0.838519	8.57E-07
5	158272130	rs3981205	T	G	0.403985	0.331666	0.73215	0.0604776	0.650573	0.823957	2.87E-07
5	158274071	rs1978706	T	C	0.401012	0.330812	0.738406	0.0603584	0.656046	0.831106	6.63E-07
5	158274491	rs17643057	A	G	0.352488	0.423298	0.741653	0.0617845	0.657882	0.836091	6.77E-07
5	158275318	chr5:158275318:I	A	AT	0.400819	0.33074	0.738758	0.0603854	0.656352	0.831509	6.84E-07
5	158275319	chr5:158275319:I	T	TA	0.404647	0.333972	0.737764	0.0605715	0.655601	0.830223	5.17E-07
5	158275601	rs2033448	T	A	0.400573	0.330403	0.73839	0.0604198	0.656016	0.831108	6.51E-07
5	158276323	rs6871291	A	G	0.400737	0.330564	0.738423	0.0604148	0.656051	0.831138	6.53E-07
5	158290225	rs61045787	C	T	0.352265	0.423462	0.740433	0.0617845	0.656791	0.834727	5.91E-07
5	158294657	chr5:158294657:I	T	TTC	0.400329	0.329969	0.737693	0.0604251	0.655385	0.830337	6.05E-07
5	158296996	rs2072495	C	T	0.353324	0.423109	0.74495	0.0617349	0.660842	0.839762	9.87E-07
5	158299860	rs9313794	G	C	0.413681	0.48877	0.737978	0.059745	0.656752	0.82925	2.82E-07
5	158300126	rs10866709	C	T	0.347181	0.419483	0.735976	0.0620553	0.652599	0.830006	3.62E-07
5	158300798	rs31196	C	A	0.474766	0.405173	0.75357	0.059199	0.671303	0.845918	1.69E-06
5	158305076	rs17543640	T	A	0.346598	0.419209	0.734911	0.062077	0.651627	0.828839	3.22E-07
5	158310631	rs929626	A	G	0.40896	0.486134	0.731402	0.0598673	0.650788	0.822002	1.27E-07
5	158310882	rs1469069	T	G	0.411613	0.488136	0.733567	0.0597614	0.652779	0.824354	1.69E-07
5	158317804	rs7737500	G	C	0.343225	0.418128	0.727247	0.0622212	0.644676	0.820395	1.32E-07
5	158318147	rs7724989	T	G	0.342984	0.417901	0.727145	0.0622357	0.644573	0.820295	1.31E-07
5	158319479	rs62385377	G	A	0.343069	0.417805	0.727707	0.0622575	0.645075	0.820924	1.39E-07
5	158319915	chr5:158319915:D	AT	A	0.342837	0.417485	0.727916	0.0622616	0.645248	0.821175	1.43E-07
5	158320876	rs1422797	T	A	0.338522	0.41248	0.728941	0.0630488	0.645933	0.822616	1.38E-07
5	158320877	rs1422798	C	G	0.33705	0.411775	0.726267	0.06309	0.64349	0.819692	1.02E-07
5	158321121	rs1422799	C	G	0.346617	0.421629	0.727709	0.0624015	0.645252	0.820703	1.17E-07
5	158332380	rs17543752	T	C	0.34104	0.416106	0.726234	0.0622497	0.643668	0.81939	1.23E-07

5	158333692	rs6556370	T	C	0.407546	0.33459	0.730975	0.0602879	0.649646	0.822486	2.49E-07
5	158343969	rs7736883	A	G	0.341567	0.416081	0.728012	0.062408	0.645269	0.821366	1.41E-07
5	158367249	rs62385385	T	A	0.341941	0.415807	0.730045	0.0623702	0.647086	0.823638	1.82E-07
5	158381512	rs1081073	T	A	0.436377	0.505998	0.755879	0.0592025	0.673144	0.848784	2.18E-06
5	158381663	rs1081071	T	C	0.34374	0.414091	0.741118	0.0623102	0.656979	0.836034	6.71E-07
5	158410081	rs12153593	C	G	0.393853	0.329299	0.755623	0.0603488	0.671125	0.850761	4.82E-06
5	158418490	rs10447210	C	T	0.393163	0.329132	0.757241	0.0603845	0.672539	0.852609	5.68E-06
5	158424391	rs10040979	G	A	0.375723	0.312745	0.756107	0.0606211	0.670782	0.852285	7.07E-06
5	158424451	rs10061082	A	G	0.375727	0.312733	0.756049	0.0606554	0.670731	0.85222	6.95E-06
5	158433339	rs67265526	T	C	0.34896	0.412493	0.763421	0.0615749	0.676986	0.860891	8.67E-06
5	158435950	rs9791127	T	C	0.376104	0.312904	0.755436	0.0608298	0.670202	0.851509	6.05E-06
5	158442930	rs6894893	T	C	0.393934	0.328758	0.753518	0.0605642	0.669251	0.848395	3.59E-06

Abbreviations: Chr, chromosome; A, coded allele; B, uncoded allele; MAF, minor allele frequency; OR, odds ratio; SE, standard error; L95, lower 95% confidence interval; U95, upper 95% confidence interval; P, *P*-value
